# Supplementary material for: Comparative Genomic Analysis of Key Oncogenic Pathways in Hepatocellular Carcinoma Among Diverse Populations
Source: Cancers (Basel). 2025 Apr 13;17(8):1309. doi: 10.3390/cancers17081309 (PMC12025884; doi:10.3390/cancers17081309)
Supplement: Supplementary file 1 [file cancers-17-01309-s001.zip › cancers-3532276-supplementary.pdf]

**Supplementary Materials:**

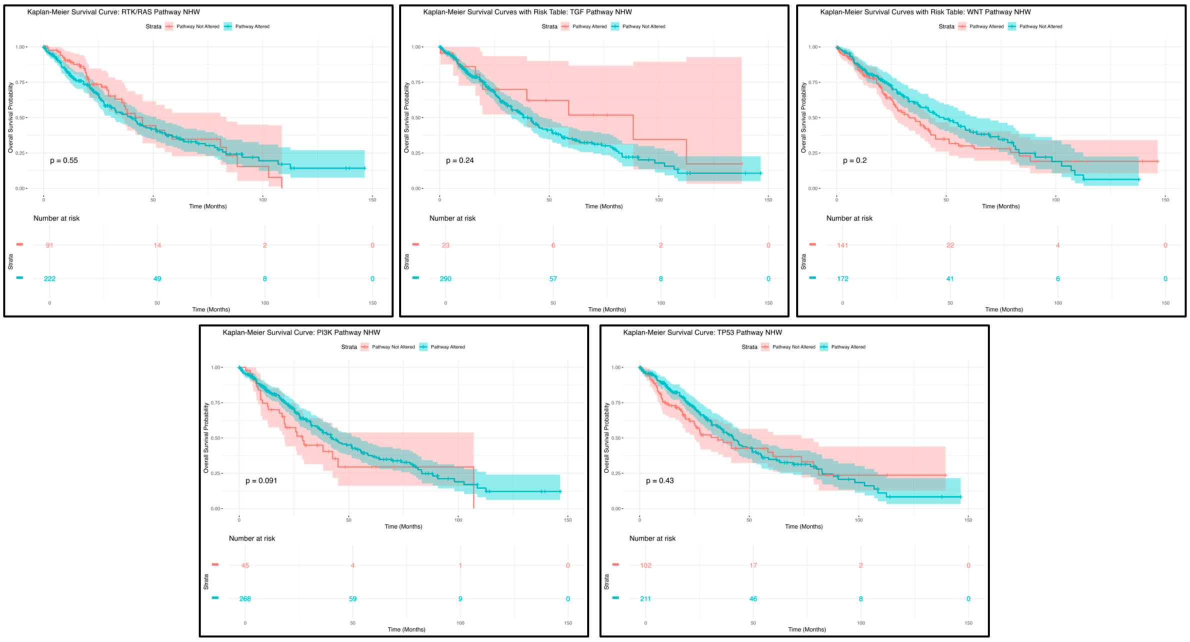


**Figure S1.** Kaplan-Meier overall survival curves for Non-Hispanic White (NHW) hepatocellular carcinoma (HCC) patients, stratified by the presence or absence of RTK/RAS (upper left), TGF-beta (upper middle), WNT (upper right), PI3K (lower left), and TP53 (lower right) pathway alterations.

**Table S1.** Alteration rates of TGF-Beta, RTK/RAS, WNT, PI3K, and TP53 pathway-related genes among Hispanic/Latino (H/L) and Non-Hispanic White (NHW) hepatocellular carcinoma (HCC) patients.

| RTK/RAS Pathway | | | |
|---|---|---|---|
| **Gene** | **H/L Samples n (%)** | **NHW Samples n (%)** | **p-value** |
| **EGFR Mutation** | | | |
| Present | 2 (2.9%) | 6 (1.3%) | 0.2669 |
| Absent | 67 (97.1%) | 472 (98.7%) | |
| **ERBB2 Mutation** | | | |
| Present | 1 (1.4%) | 4 (0.8%) | 0.4918 |
| Absent | 68 (98.6%) | 474 (99.2%) | |
| **ERBB4 Mutation** | | | |
| Present | 4 (5.8%) | 11 (2.3%) | 0.1077 |
| Absent | 65 (94.2%) | 467 (97.7%) | |
| **MET Mutation** | | | |
| Present | 2 (2.9%) | 7 (1.5%) | 0.3168 |
| Absent | 67 (97.1%) | 471 (98.5%) | |
| **PDGFRA Mutation** | | | |
| Present | 0 (0.0%) | 7 (1.5%) | 0.604 |
| Absent | 69 (100.0%) | 471 (98.5%) | |
| **FGFR1 Mutation** | | | |

| | | | |
|---|---|---|---|
| Present | 2 (2.9%) | 3 (0.6%) | 0.1219 |
| Absent | 67 (97.1%) | 475 (99.4%) | |
| **FGFR2 Mutation** | | | |
| Present | 2 (2.9%) | 3 (0.6%) | 0.1219 |
| Absent | 67 (97.1%) | 475 (99.4%) | |
| **FGFR3 Mutation** | | | |
| Present | 1 (1.4%) | 4 (0.8%) | 0.4918 |
| Absent | 68 (98.6%) | 474 (99.2%) | |
| **FGFR4 Mutation** | | | |
| Present | 3 (4.3%) | 3 (0.6%) | **0.02906** |
| Absent | 66 (95.7%) | 475 (99.4%) | |
| **KIT Mutation** | | | |
| Present | 2 (2.9%) | 12 (2.5%) | 0.693 |
| Absent | 67 (97.1%) | 466 (97.5%) | |
| **IGF1R Mutation** | | | |
| Present | 5 (7.2%) | 14 (2.9%) | 0.07798 |
| Absent | 64 (92.8%) | 464 (97.1%) | |
| **RET Mutation** | | | |
| Present | 0 (0.0%) | 8 (1.7%) | 0.6044 |
| Absent | 69 (100.0%) | 470 (98.3%) | |
| **ROS1 Mutation** | | | |
| Present | 1 (1.4%) | 11 (2.3%) | 1 |
| Absent | 68 (98.6%) | 467 (97.7%) | |
| **ALK Mutation** | | | |
| Present | 1 (1.4%) | 14 (2.9%) | 0.7063 |
| Absent | 68 (98.6%) | 464 (97.1%) | |
| **FLT3 Mutation** | | | |
| Present | 0 (0.0%) | 4 (0.8%) | 1 |
| Absent | 69 (100.0%) | 474 (99.2%) | |
| **NTRK1 Mutation** | | | |
| Present | 1 (1.4%) | 10 (2.1%) | 1 |
| Absent | 68 (98.6%) | 468 (97.9%) | |
| **NTRK2 Mutation** | | | |
| Present | 0 (0.0%) | 10 (2.1%) | 0.6225 |
| Absent | 69 (100.0%) | 468 (97.9%) | |
| **CBL Mutation** | | | |
| Present | 1 (1.4%) | 8 (1.7%) | 1 |
| Absent | 68 (98.6%) | 470 (98.3%) | |
| **ERRFI1 Mutation** | | | |
| Present | 0 (0.0%) | 8 (1.7%) | 0.6044 |
| Absent | 69 (100.0%) | 470 (98.3%) | |

| | | | |
|---|---|---|---|
| **SOS1 Mutation** | | | |
| Present | 1 (1.4%) | 4 (0.8%) | 0.4918 |
| Absent | 68 (98.6%) | 474 (99.2%) | |
| **NF1 Mutation** | | | |
| Present | 2 (2.9%) | 11 (2.3%) | 0.6734 |
| Absent | 67 (97.1%) | 467 (97.7%) | |
| **RASA1 Mutation** | | | |
| Present | 0 (0.0%) | 6 (1.3%) | 1 |
| Absent | 69 (100.0%) | 472 (98.7%) | |
| **PTPN11 Mutation** | | | |
| Present | 2 (2.9%) | 4 (0.8%) | 0.1683 |
| Absent | 67 (97.1%) | 474 (99.2%) | |
| **KRAS Mutation** | | | |
| Present | 1 (1.4%) | 12 (2.5%) | 1 |
| Absent | 68 (98.6%) | 466 (97.5%) | |
| **HRAS Mutation** | | | |
| Present | 0 (0.0%) | 1 (0.2%) | 1 |
| Absent | 69 (100.0%) | 477 (99.8%) | |
| **NRAS Mutation** | | | |
| Present | 2 (2.9%) | 6 (1.3%) | 0.2669 |
| Absent | 67 (97.1%) | 472 (98.7%) | |
| **RIT1 Mutation** | | | |
| Present | 0 (0.0%) | 0 (0.0%) | 1 |
| Absent | 69 (100.0%) | 478 (100.0%) | |
| **ARAF Mutation** | | | |
| Present | 0 (0.0%) | 6 (1.3%) | 1 |
| Absent | 69 (100.0%) | 472 (98.7%) | |
| **BRAF Mutation** | | | |
| Present | 0 (0.0%) | 6 (1.3%) | 1 |
| Absent | 69 (100.0%) | 472 (98.7%) | |
| **RAF1 Mutation** | | | |
| Present | 1 (1.4%) | 3 (0.6%) | 0.4178 |
| Absent | 68 (98.6%) | 475 (99.4%) | |
| **RAC1 Mutation** | | | |
| Present | 0 (0.0%) | 0 (0.0%) | 1 |
| Absent | 69 (100.0%) | 478 (100.0%) | |
| **MAPK1 Mutation** | | | |
| Present | 0 (0.0%) | 2 (0.4%) | 1 |
| Absent | 69 (100.0%) | 476 (99.6%) | |
| **MAP2K1 Mutation** | | | |
| Present | 0 (0.0%) | 3 (0.6%) | 1 |

| | | | |
|---|---|---|---|
| Absent | 69 (100.0%) | 475 (99.4%) | |
| **MAP2K2 Mutation** | | | |
| Present | 0 (0.0%) | 3 (0.6%) | 1 |
| Absent | 69 (100.0%) | 475 (99.4%) | |
| **TGF-Beta Pathway** | | | |
| **Gene** | **H/L Samples n (%)** | **NHW Samples n (%)** | **p-value** |
| **ACVR2A Mutation** | | | |
| Present | 0 (0.0%) | 7 (1.5%) | 0.604 |
| Absent | 69 (100.0%) | 471 (98.5%) | |
| **ACVR2B Mutation** | | | |
| Present | 0 (0.0%) | 1 (0.2%) | 1 |
| Absent | 69 (100.0%) | 477 (99.8%) | |
| **SMAD2 Mutation** | | | |
| Present | 0 (0.0%) | 3 (0.6%) | 1 |
| Absent | 69 (100.0%) | 475 (99.4%) | |
| **SMAD3 Mutation** | | | |
| Present | 0 (0.0%) | 6 (1.3%) | 1 |
| Absent | 69 (100.0%) | 472 (98.7%) | |
| **SMAD4 Mutation** | | | |
| Present | 0 (0.0%) | 4 (0.8%) | 1 |
| Absent | 69 (100.0%) | 474 (99.2%) | |
| **TGFBR1 Mutation** | | | |
| Present | 0 (0.0%) | 3 (0.6%) | 1 |
| Absent | 69 (100.0%) | 475 (99.4%) | |
| **TGFBR2 Mutation** | | | |
| Present | 2 (2.9%) | 2 (0.4%) | 0.07951 |
| Absent | 67 (97.1%) | 476 (99.6%) | |
| **WNT Pathway** | | | |
| **Gene** | **H/L Samples n (%)** | **NHW Samples n (%)** | **p-value** |
| **AMER1 Mutation** | | | |
| Present | 1 (1.4%) | 5 (1.0%) | 0.5565 |
| Absent | 68 (98.6%) | 473 (99.0%) | |
| **APC Mutation** | | | |
| Present | 4 (5.8%) | 23 (4.8%) | 0.7642 |
| Absent | 65 (94.2%) | 455 (95.2%) | |
| **AXIN1 Mutation** | | | |
| Present | 6 (8.7%) | 18 (3.8%) | 0.12 |
| Absent | 63 (91.3%) | 460 (96.2%) | |
| **AXIN2 Mutation** | | | |

| | | | |
|---|---|---|---|
| Present | 1 (1.4%) | 7 (1.5%) | 1 |
| Absent | 68 (98.6%) | 471 (98.5%) | |
| **CTNNB1 Mutation** | | | |
| Present | 22 (31.9%) | 155 (32.4%) | 1 |
| Absent | 47 (68.1%) | 323 (67.6%) | |
| **DKK1 Mutation** | | | |
| Present | 0 (0.0%) | 0 (0.0%) | 1 |
| Absent | 69 (100.0%) | 478 (100.0%) | |
| **DKK2 Mutation** | | | |
| Present | 1 (1.4%) | 0 (0.0%) | 0.1261 |
| Absent | 68 (98.6%) | 478 (100.0%) | |
| **DKK3 Mutation** | | | |
| Present | 0 (0.0%) | 1 (0.2%) | 1 |
| Absent | 69 (100.0%) | 477 (99.8%) | |
| **DKK4 Mutation** | | | |
| Present | 0 (0.0%) | 0 (0.00%) | 1 |
| Absent | 69 (100.0%) | 478 (100.00%) | |
| **GSK3B Mutation** | | | |
| Present | 1 (1.4%) | 2 (0.4%) | 0.3332 |
| Absent | 68 (98.6%) | 476 (99.6%) | |
| **LRP5 Mutation** | | | |
| Present | 0 (0.0%) | 3 (0.6%) | 1 |
| Absent | 69 (100.0%) | 475 (99.4%) | |
| **LRP6 Mutation** | | | |
| Present | 0 (0.0%) | 2 (0.4%) | 1 |
| Absent | 69 (100.0%) | 476 (99.6%) | |
| **RNF43 Mutation** | | | |
| Present | 0 (0.0%) | 5 (1.0%) | 1 |
| Absent | 69 (100.0%) | 473 (99.0%) | |
| **SFRP1 Mutation** | | | |
| Present | 0 (0.0%) | 0 (0.0%) | 1 |
| Absent | 69 (100.0%) | 478 (100.0%) | |
| **SFRP2 Mutation** | | | |
| Present | 0 (0.0%) | 1 (0.2%) | 1 |
| Absent | 69 (100.0%) | 477 (99.8%) | |
| **SFRP3 Mutation** | | | |
| Present | 0 (0.0%) | 0 (0.0%) | 1 |
| Absent | 69 (100.0%) | 478 (100.0%) | |
| **SFRP4 Mutation** | | | |
| Present | 0 (0.0%) | 0 (0.0%) | 1 |
| Absent | 69 (100.0%) | 478 (100.0%) | |

| | | | |
|---|---|---|---|
| **SFRP5 Mutation** | | | |
| Present | 0 (0.0%) | 0 (0.0%) | 1 |
| Absent | 69 (100.0%) | 478 (100.0%) | |
| **TCF7 Mutation** | | | |
| Present | 0 (0.0%) | 2 (0.4%) | 1 |
| Absent | 69 (100.0%) | 476 (99.6%) | |
| **TCF7L1 Mutation** | | | |
| Present | 1 (1.4%) | 0 (0.0%) | 0.1261 |
| Absent | 68 (98.6%) | 478 (100.0%) | |
| **TCF7L2 Mutation** | | | |
| Present | 0 (0.0%) | 6 (1.3%) | 1 |
| Absent | 69 (100.0%) | 472 (98.7%) | |
| **TLE1 Mutation** | | | |
| Present | 1 (1.4%) | 3 (0.6%) | 0.4178 |
| Absent | 68 (98.6%) | 475 (99.4%) | |
| **TLE2 Mutation** | | | |
| Present | 0 (0.0%) | 1 (0.2%) | 1 |
| Absent | 69 (100.0%) | 477 (99.8%) | |
| **TLE3 Mutation** | | | |
| Present | 0 (0.0%) | 1 (0.2%) | 1 |
| Absent | 69 (100.0%) | 477 (99.8%) | |
| **TLE4 Mutation** | | | |
| Present | 0 (0.0%) | 3 (0.6%) | 1 |
| Absent | 69 (100.0%) | 475 (99.4%) | |
| **WIF1 Mutation** | | | |
| Present | 0 (0.0%) | 0 (0.0%) | 1 |
| Absent | 69 (100.0%) | 478 (100.0%) | |
| **PI3K Pathway** | | | |
| **Gene** | **H/L Samples n (%)** | **NHW Samples n (%)** | **p-value** |
| **PTEN Mutation** | | | |
| Present | 3 (4.3%) | 7 (1.5%) | 0.1202 |
| Absent | 66 (95.7%) | 471 (98.5%) | |
| **PIK3R1 Mutation** | | | |
| Present | 0 (0.0%) | 3 (0.6%) | 1 |
| Absent | 69 (100.0%) | 475 (99.4%) | |
| **PIK3R2 Mutation** | | | |
| Present | 0 (0.0%) | 2 (0.4%) | 1 |
| Absent | 69 (100.0%) | 476 (99.6%) | |
| **PIK3R3 Mutation** | | | |
| Present | 0 (0.0%) | 0 (0.0%) | 1 |

| | | | |
|---|---|---|---|
| Absent | 69 (100.0%) | 478 (100.0%) | |
| **PIK3CA Mutation** | | | |
| Present | 2 (2.9%) | 9 (1.9%) | 0.6374 |
| Absent | 67 (97.1%) | 469 (98.1%) | |
| **INPP4B Mutation** | | | |
| Present | 3 (4.3%) | 5 (1.0%) | 0.06749 |
| Absent | 66 (95.7%) | 473 (99.0%) | |
| **AKT1 Mutation** | | | |
| Present | 1 (1.4%) | 1 (0.2%) | 0.2366 |
| Absent | 68 (98.6%) | 477 (99.8%) | |
| **AKT2 Mutation** | | | |
| Present | 0 (0.0%) | 7 (1.5%) | 0.604 |
| Absent | 69 (100.0%) | 471 (98.5%) | |
| **AKT3 Mutation** | | | |
| Present | 0 (0.0%) | 4 (0.8%) | 1 |
| Absent | 69 (100.0%) | 474 (99.2%) | |
| **PPP2R1A Mutation** | | | |
| Present | 1 (1.4%) | 6 (1.3%) | 1 |
| Absent | 68 (98.6%) | 472 (98.7%) | |
| **TSC1 Mutation** | | | |
| Present | 2 (2.9%) | 11 (2.3%) | 0.6734 |
| Absent | 67 (97.1%) | 467 (97.7%) | |
| **TSC2 Mutation** | | | |
| Present | 3 (4.3%) | 20 (4.2%) | 1 |
| Absent | 66 (95.7%) | 458 (95.8%) | |
| **STK11 Mutation** | | | |
| Present | 1 (1.4%) | 3 (0.6%) | 0.4178 |
| Absent | 68 (98.6%) | 475 (99.4%) | |
| **RHEB Mutation** | | | |
| Present | 0 (0.0%) | 1 (0.2%) | 1 |
| Absent | 69 (100.0%) | 477 (99.8%) | |
| **RICTOR Mutation** | | | |
| Present | 0 (0.0%) | 7 (1.5%) | 0.604 |
| Absent | 69 (100.0%) | 471 (98.5%) | |
| **MTOR Mutation** | | | |
| Present | 2 (2.9%) | 14 (2.9%) | 1 |
| Absent | 67 (97.1%) | 464 (97.1%) | |
| **RPTOR Mutation** | | | |
| Present | 1 (1.4%) | 5 (1.0%) | 0.5565 |
| Absent | 68 (98.6%) | 473 (99.0%) | |
| **TP53 Pathway** | | | |

| Gene | H/L Samples n (%) | NHW Samples n (%) | p-value |
|---|---|---|---|
| **TP53 Mutation** | | | |
| Present | 24 (34.8%) | 131 (27.4%) | 0.2592 |
| Absent | 45 (65.2%) | 347 (72.6%) | |
| **MDM2 Mutation** | | | |
| Present | 1 (1.4%) | 0 (0.0%) | 0.1261 |
| Absent | 68 (98.6%) | 478 (100.0%) | |
| **MDM4 Mutation** | | | |
| Present | 0 (0.0%) | 3 (0.6%) | 1 |
| Absent | 69 (100.0%) | 475 (99.4%) | |
| **CDKN2A Mutation** | | | |
| Present | 2 (2.9%) | 14 (2.9%) | 1 |
| Absent | 67 (97.1%) | 464 (97.1%) | |
| **ATM Mutation** | | | |
| Present | 1 (1.4%) | 24 (5.0%) | 0.3482 |
| Absent | 68 (98.6%) | 454 (95.0%) | |
| **CHEK2 Mutation** | | | |
| Present | 1 (1.4%) | 6 (1.3%) | 1 |
| Absent | 68 (98.6%) | 472 (98.7%) | |
| **RPS6KA3 Mutation** | | | |
| Present | 1 (1.4%) | 8 (1.7%) | 1 |
| Absent | 68 (98.6%) | 470 (98.3%) | |

**Table S2.** Nature of gene mutations within genes TGFBR2, IGF1R, FGFR4, INPP4B in Non-Hispanic White HCC patients. Mutation types include frame shift deletions, frame shift insertions, missense mutations, nonsense mutations, splice site mutations, and translation start site mutations.

| | NHW Samples | | | |
|---|---|---|---|---|
| | Frame Shift Deletion | Missense Mutation | Nonsense Mutation | Splice Region |
| TGFBR2 | 0% | 50% | 0% | 50% |
| IGF1R | 7% | 80% | 7% | 7% |
| FGFR4 | 0% | 100% | 0% | 0% |
| INPP4B | 0% | 100% | 0% | 0% |
